# Dietary Supplementation with Soluble Plantain Non-Starch Polysaccharides Inhibits Intestinal Invasion of *Salmonella* Typhimurium in the Chicken

**DOI:** 10.1371/journal.pone.0087658

**Published:** 2014-02-03

**Authors:** Bryony N. Parsons, Paul Wigley, Hannah L. Simpson, Jonathan M. Williams, Suzie Humphrey, Anne-Marie Salisbury, Alastair J. M. Watson, Stephen C. Fry, David O'Brien, Carol L. Roberts, Niamh O'Kennedy, Åsa V. Keita, Johan D. Söderholm, Jonathan M. Rhodes, Barry J. Campbell

**Affiliations:** 1 Gastroenterology, Institute of Translational Medicine, University of Liverpool, Liverpool, United Kingdom; 2 Infection Biology, Institute of Infection and Global Health, University of Liverpool, Leahurst, United Kingdom; 3 Norwich Medical School, University of East Anglia, Norwich Research Park, Norwich, United Kingdom; 4 The Edinburgh Cell Wall Group, Institute of Molecular Plant Sciences, University of Edinburgh, Edinburgh, United Kingdom; 5 Provexis plc, c/o Rowett Institute of Nutrition and Health, Aberdeen, United Kingdom; 6 Clinical and Experimental Medicine, Division of Surgery, Faculty of Health Sciences, Linköping University, Linköping, Sweden; University of Osnabrueck, Germany

## Abstract

Soluble fibres (non-starch polysaccharides, NSP) from edible plants but particularly plantain banana (*Musa* spp.), have been shown *in vitro* and *ex vivo* to prevent various enteric pathogens from adhering to, or translocating across, the human intestinal epithelium, a property that we have termed contrabiotic. Here we report that dietary plantain fibre prevents invasion of the chicken intestinal mucosa by *Salmonella*. *In vivo* experiments were performed with chicks fed from hatch on a pellet diet containing soluble plantain NSP (0 to 200 mg/d) and orally infected with *S*.Typhimurium 4/74 at 8 d of age. Birds were sacrificed 3, 6 and 10 d post-infection. Bacteria were enumerated from liver, spleen and caecal contents. *In vitro* studies were performed using chicken caecal crypts and porcine intestinal epithelial cells infected with *Salmonella enterica* serovars following pre-treatment separately with soluble plantain NSP and acidic or neutral polysaccharide fractions of plantain NSP, each compared with saline vehicle. Bacterial adherence and invasion were assessed by gentamicin protection assay. *In vivo* dietary supplementation with plantain NSP 50 mg/d reduced invasion by *S.*Typhimurium, as reflected by viable bacterial counts from splenic tissue, by 98.9% (95% CI, 98.1–99.7; *P*<0.0001). *In vitro* studies confirmed that plantain NSP (5–10 mg/ml) inhibited adhesion of *S.*Typhimurium 4/74 to a porcine epithelial cell-line (73% mean inhibition (95% CI, 64–81); *P*<0.001) and to primary chick caecal crypts (82% mean inhibition (95% CI, 75–90); *P*<0.001). Adherence inhibition was shown to be mediated via an effect on the epithelial cells and Ussing chamber experiments with *ex-vivo* human ileal mucosa showed that this effect was associated with increased short circuit current but no change in electrical resistance. The inhibitory activity of plantain NSP lay mainly within the acidic/pectic (homogalacturonan-rich) component. Supplementation of chick feed with plantain NSP was well tolerated and shows promise as a simple approach for reducing invasive salmonellosis.

## Introduction

*Salmonella enterica* infection in humans is associated with self-limiting diarrhoea, fever, and abdominal pains [1], [2]. In England and Wales, 9,685 human cases of *Salmonella* infection were confirmed in 2010, the most commonly isolated serovars *Salmonella enterica* Enteritidis and *Salmonella enterica* Typhimurium [3]. Poultry-related products are one of the major sources of *Salmonella* infection for humans [2], [4], [5]. *Salmonella* also causes considerable worldwide economic loss through chicken mortality, primarily caused by the avian-adapted serovars *S*. Gallinarum and *S*. Pullorum [6], [7]. Pigs are also a frequent source of zoonotic infection [8]. The prevalence of *Salmonella* in pigs varies from 7.9 to 30% depending upon the country [8]–[10].

Vaccination has been successfully used to reduce *Salmonella* in laying hens, however the cost and practicalities make vaccines unsuitable for use in broilers. The use of therapeutic antimicrobials against *Salmonella* is increasingly limited in poultry production due to problems with the emergence of resistant epidemic isolates [11]. Since there are no vaccines to prevent salmonellosis, or indeed other food-borne bacteria in humans, there is a clear need for an alternative preventative approach.

Various substances have been investigated for their potentially inhibitory effects on *Salmonella* infection and faecal shedding, including butyrate [12], honey [13], acidification of feed using lactic, formic and acetic acid [14], [15], glutamine [16], glycopeptides derived from soybeans [17], and partially digested whey protein [18]. Butyrate showed promising results for reducing *Salmonella* colonisation in chickens *in vivo* via up-regulation of host defence peptides [12]. Acidified feed also inhibited *Salmonella* shedding in pigs, [15], but other interventions showed limitations, such as possible cytotoxicity to cell monolayers at high concentrations [18], or attenuated effects *in vivo* [14], [16].

We have previously demonstrated that soluble NSP from plantain banana (*Musa* spp.), inhibits the adhesion of *Escherichia coli* to, and invasion into, human intestinal epithelial cells [19] and translocation across specialised microfold (M)-cells of the follicle-associated epithelium (FAE) cultured *in vitro* [19], [20]. Subsequently we recently described that soluble plantain NSP was also able to block adhesion of various enteric gut pathogens to the human intestinal epithelial cell-line Caco2, including *S*. Typhimurium, *Shigella sonnei*, *Clostridium difficile* and diarrheagenic enterotoxigenic *E. coli* (ETEC) [21], with the only exception being enteropathogenic *E. coli*, where plantain NSP did not block bacterial adherence [21]. In the same study, soluble plantain NSP was also shown to block translocation of *S*. Typhimurium across M-cells in culture and *ex vivo* human ileal FAE mounted in Ussing chambers [21]. Other soluble plant NSP preparations, such as broccoli NSP, have also showed significant ability to block pathogen-epithelium interaction [20].

We therefore speculated that soluble plantain NSP may also inhibit *Salmonella* in an un-manipulated animal model. To investigate this we performed additional *in vitro* experiments to assess the inhibitory action of plantain NSP on *Salmonella* interaction with porcine-derived intestinal epithelial cells (B1OXI cell-line) and primary chicken caecal crypts. We also conducted an *in vivo* study to investigate the potential protective effect of dietary supplementation with soluble plantain NSP in a model of invasive salmonellosis in inbred White Leghorn Line 0 chicks.

## Results

### Supplementation of chick feed with soluble plantain NSP reduces *S.* Typhimurium 4/74 translocation to the spleen *in vivo*

**Figure 1:**
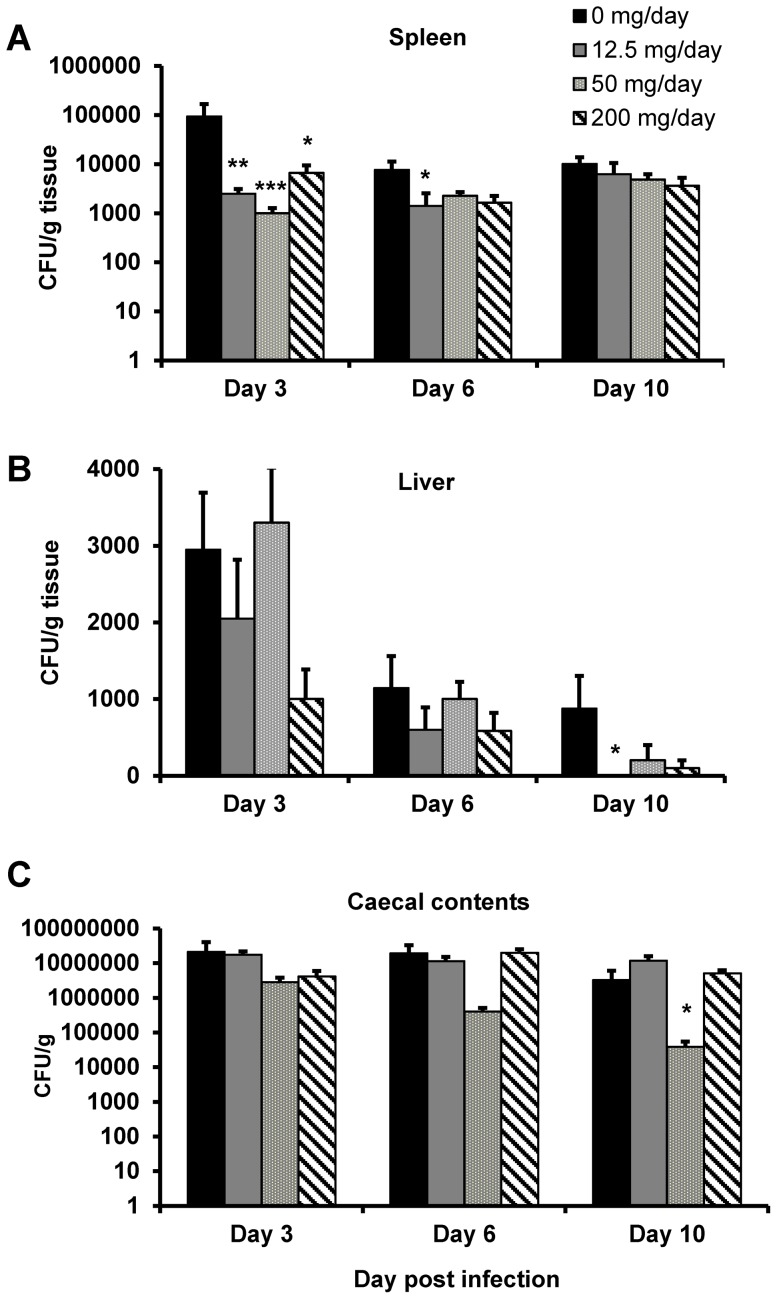
Dietary supplementation with soluble plantain NSP reduces chick salmonellosis *in vivo.* (**A**) Following infection of 8 day-old inbred specified pathogen-free White Leghorn Line 0 chicks with *S.* Typhimurium 4/74, soluble plantain NSP supplementation of a commercial pellet feed significantly reduced bacterial numbers found in the spleen 3 d post-infection. (**B**) Supplementation with soluble plantain NSP had little significant effect on presence of *S.* Typhimurium CFU cultured from the liver, excepting at 10 d post infection. (**C**) Total CFU observed within the caecal lumen were relatively unchanged at all doses of plantain NSP supplementation compared to birds fed a control diet. Significant differences from control (non-supplemented NSP) diet, * *P*<0.05; ** *P*<0.01; *** *P*<0.0001 Kruskal-Wallis (N = 4–7 birds, n = 2 replicates).

In chicks ingesting a custom-made commercial pellet diet supplemented with soluble plantain NSP there was significant reduction observed in the translocation of *S.* Typhimurium 4/74 across the chick gut. The most profound effect observed, with all three doses of ingested soluble plantain NSP (12.5, 50 and 200 mg/d; all *P*<0.05 Kruskal-Wallis), was a significant reduction in Salmonellae found in the splenic tissue (e.g. CFUs reduced in splenic tissue by 98.9% (95% CI, 98.1–99.7) 3 d post-infection, in birds on 50 mg/d plantain NSP (N = 5 birds, n = 2 replicates) compared to those birds on a non-supplemented NSP control diet (N = 5, n = 2; *P*<0.0001); **Figure 1A**. Supplementation with soluble plantain NSP had little effect on presence of *S.* Typhimurium CFU cultured from the liver, excepting at 10 d post infection (*P*<0.05), however bacterial counts in the liver were orders of magnitude lower than spleen counts. There was no significant effect of plantain NSP supplementation on the total CFU observed within the caecal lumen at all doses of plantain NSP supplementation, with the exception of some reduction at day ten with one dose only, compared to birds fed a control diet (see **Figure 1B and 1C**).

Consistent with *S*. Typhimurium infection in birds of this age, mild inflammation of caecal tissue was seen in all infected birds receiving the standard commercial pellet feed (i.e. not receiving plantain NSP). Livers from infected birds exhibited mild periportal and multifocal lymphoplasmacytic, histiocytic, and heterophilic infiltrates, with variable single cell hepatocellular necrosis. Focal necrosis was only seen in 3/36 livers examined, two of which were in control feed birds, whilst the other was in the group fed 50 mg/d plantain NSP. No significant abnormalities were observed in spleen and ileal tissue sections taken from all treatment groups.

### Further experiments to clarify the mechanism of inhibition of *Salmonella* spp. interaction with intestinal epithelia: *in vitro* studies

**Figure 2:**
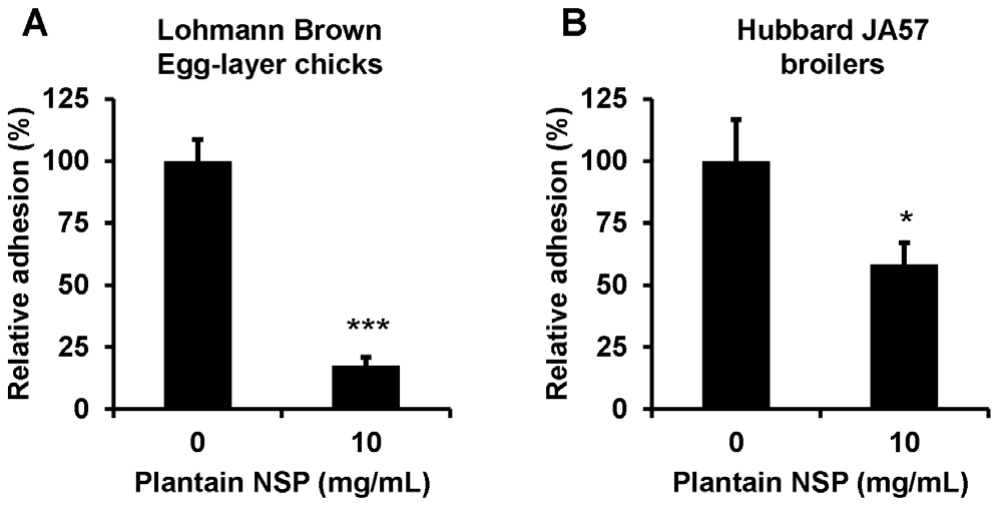
Soluble plantain NSP reduces adherence of *S*. Typhimurium 4/74 to primary caecal crypts. Soluble plantain NSP (10 mg/mL) reduced adherence of *S*. Typhimurium 4/74 to primary chick caecal crypts isolated from both (**A**) 14-day old Lohmann Brown Classic egg-layers (N = 4 experiments, n = 3 replicates; *** *P*<0.001 Mann Whitney U) and (**B**) 33-day old Hubbard JA57 broiler chickens (N = 3, n = 2; * *P* = 0.05 Mann Whitney U).

Soluble plantain NSP, at 10 mg/mL, reduced adhesion of *S*. Typhimurium 4/74 to primary caecal crypts isolated from 14-day old Lohmann Brown Classic egg-layer chicks (mean reduction of 82% (95% CI, 75–90), N = 4 birds, n = 3 replicates; *P*<0.001 Mann Whitney U test). Likewise, plantain NSP reduced strain 4/74 adherence to caecal crypts from 33-day old Hubbard JA57 broiler chickens by 42% (95% CI, 19–64), N = 3, n = 2; *P* = 0.05 (see **Figure 2**). No significant effects on crypt viability were observed with either soluble plantain NSP pre-treatment nor during the 90 min infection with *S.* Typhimurium 4/74 as assessed by adenylate kinase release into the culture medium (with levels within 90–98% of vehicle-treated control cells).

Plantain NSP at 10 mg/mL also reduced adhesion of *S.* Typhimurium 4/74 (as used in the *in vivo* infection studies) to human Caco2 cells (56% (95% CI, 46–65) reduction in adhesion (N = 3, n = 4) albeit to a lesser extent than that seen for *S*. Typhimurium LT2-infected human Caco2 cells (81% (95% CI, 65–98)); both *P*<0.001 Kruskal-Wallis; see **File S1**.

**Figure 3:**
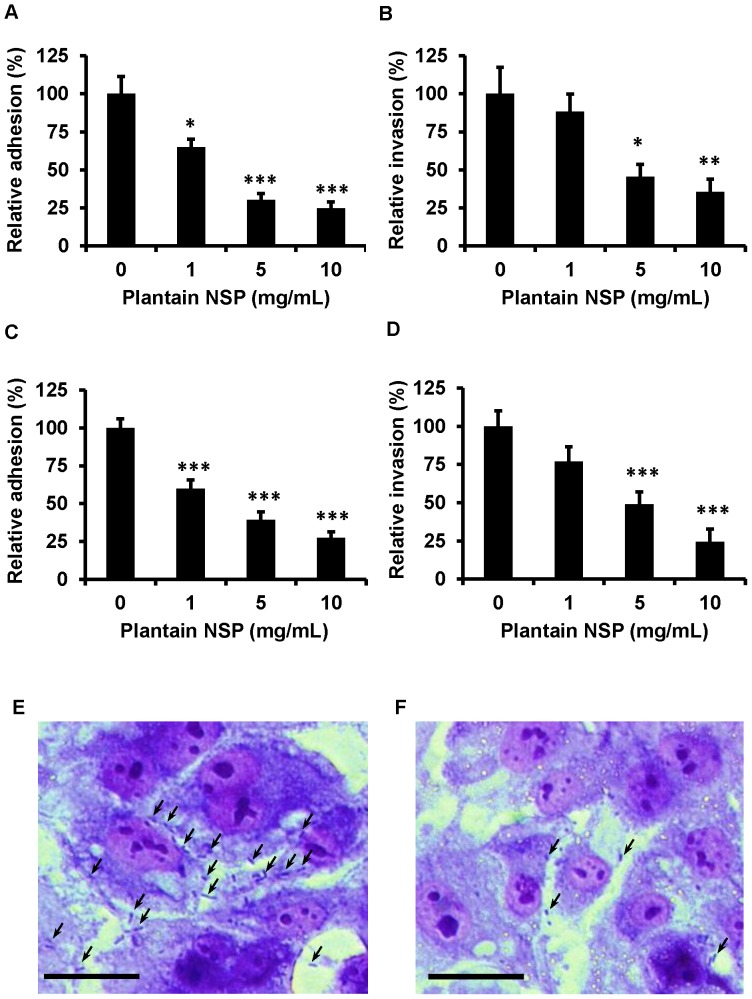
Soluble plantain NSP inhibits adhesion of *S*. Typhimurium to the porcine B1OXI enterocyte cell-line *in vitro*. Pre-treatment (30 min) with soluble plantain NSP, dose-dependently blocked adhesion of (**A**) *S*. Typhimurium LT2 and (**B**) *S*. Typhimurium 4/74 to B1OXI cells (N = 3 experiments, each with n = 4 replicates); * *P*<0.05, ** *P*<0.01, *** *P*<0.001, Kruskal-Wallis. Invasion of (**C**) *S.* Typhimurium LT2 and (**D**) *S.* Typhimurium 4/74 into B1OXI cells was also blocked by plantain NSP. Data (mean ± SEM) expressed relative to adherence (or invasion) of vehicle-treated control (100%). Light microscopy of Giemsa-stained B1OXI cells and *S*. Typhimurium 4/74 in absence (**E**) or presence (**F**) of 10 mg/mL soluble plantain NSP. Arrows indicate bacteria.

Pre-treatment of the porcine B1OXI enterocyte-like cell-line with soluble plantain NSP also significantly inhibited adhesion and invasion of *S.* Typhimurium LT2 in a dose-dependent manner. Similar results were observed for plantain NSP in blockade of *S.* Typhimurium 4/74 to porcine enterocytes (**Figure 3**). Peak reduction in both adhesion to, and invasion of B1OXI cells, was observed at concentrations of 10 mg/mL soluble plantain NSP; e.g. mean reductions in *S.* Typhimurium adherence compared to vehicle-treated control were 75% (95% CI, 66–84) and 73% (95% CI, 64–81) for strains LT2 and 4/74 respectively (N = 3, n = 4; both *P*<0.01 Kruskal-Wallis). In addition, soluble plantain NSP also significantly blocked adhesion of *S.* Enteritidis, another key *Salmonella enterica* serovar relevant to production animals, with an 80% reduction (95% CI, 73–87) seen using 10 mg/mL plantain NSP (N = 3, n = 4; *P*<0.001 Mann Whitney U test), see **File S2**. Invasion of *S.* Enteritidis to B1OXI cells was not however significantly reduced following pre-treatment with plantain NSP.

As per previous studies [20], [21], treatment of epithelial cells with plantain NSP generated no significant release of adenylate kinase to the medium indicating a lack of cytotoxicity. This was also confirmed by Giemsa microscopy (see **Figure 3**). Likewise, as per previous studies [20], [21], no bacteriocidal effect of plantain NSP was seen on Salmonellae.

### The effects of soluble plantain NSP on epithelial adherence of *Salmonella* spp. are mediated via an effect on the epithelium that is associated with a marked increase in short-circuit current

**Figure 4:**
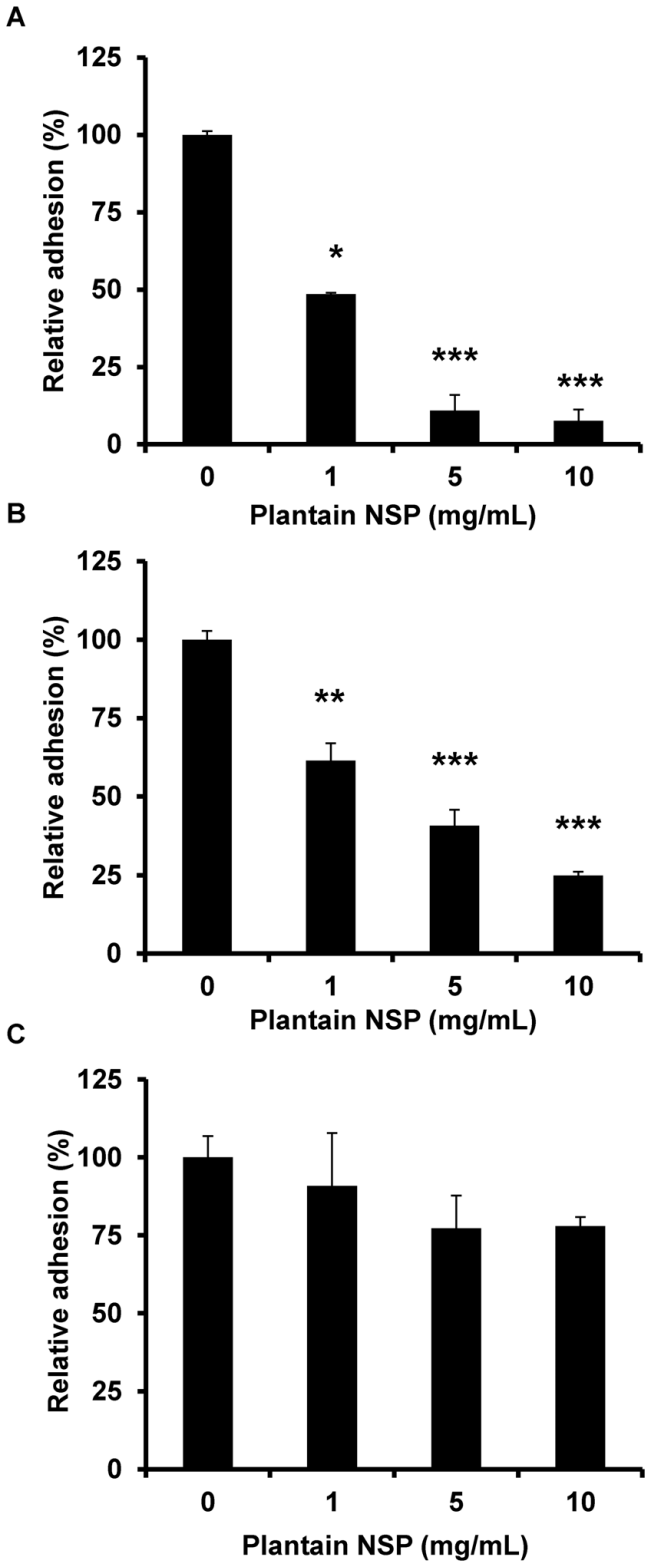
Soluble plantain NSP acts on the epithelium to block interaction of *S*. Typhimurium LT2. Plantain NSP (5 mg/mL) blockade of adhesion of *S*. Typhimurium LT2 to B1OXI cells under different pre-treatment conditions. (**A**) Standard pre-treatment of cell monolayers with soluble plantain NSP (30 min), followed by infection for 90 min. (**B**) Pre-treatment of cell monolayers with soluble plantain NSP (30 min), followed by removal from monolayers using three sterile PBS washes prior to infection for 90 min. (**C**) Pre-treatment of bacteria with plantain NSP for 30 min, followed by centrifugation, re-suspension of bacteria in antibiotic free media and infection for 90 min. Data (mean ± SEM) expressed relative to adherence of vehicle-treated control (100%); n = 3; ** *P*<0.01, *** *P*<0.001, Kruskal-Wallis.

In additional experiments using B1OXI cells, we were able to demonstrate that plantain NSP blockade of *Salmonella* adhesion to cell monolayers acts primarily through action on the epithelium. When plantain NSP (5 mg/mL) was added to monolayers 30 min prior to infection, then removed by three washes with sterile PBS (1 min each; at 37°C), levels of adherent *Salmonella* were observed to be significantly reduced (59.2±5.0% inhibition compared to untreated control; N = 1, n = 3; *P*<0.001 Kruskal-Wallis), albeit lower than that seen in experiments where plantain NSP was added to cells for 30 min without removal before infection (89±5.0% inhibition; N = 1, n = 3; P<0.001); see **Figure 4**. In contrast, plantain NSP added to bacteria for 30 min, and then removed by centrifugation before re-suspension of bacteria in antibiotic free media and inoculation of B1OXI cell monolayers, resulted in much less inhibition (22.8±10.5%) compared to untreated control (100%); **Figure 4**.

**Figure 5:**
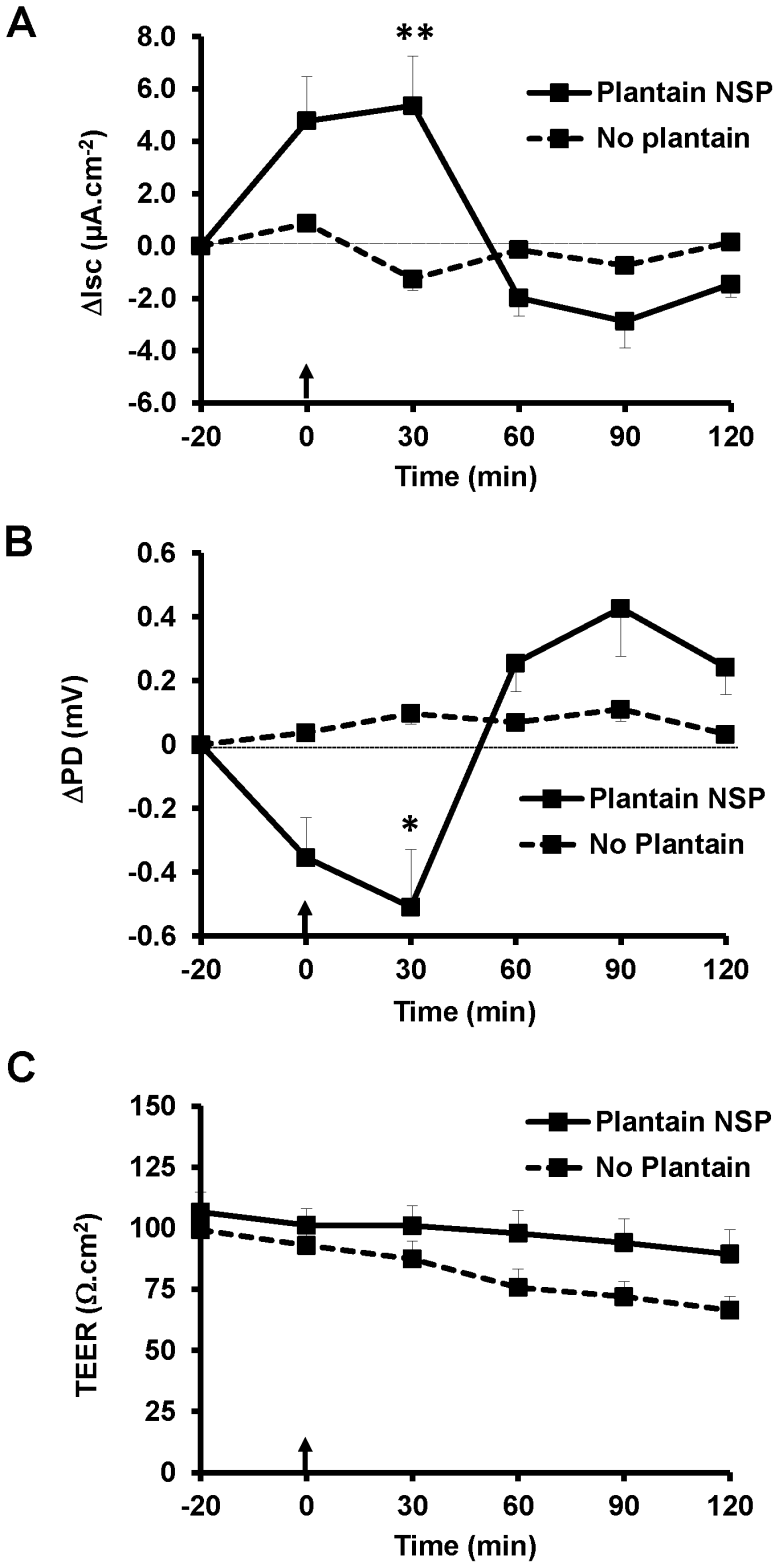
Soluble plantain NSP increases the transmucosal short circuit current of *ex vivo* human ileal follicle-associated epithelium. Plantain NSP (5 mg/mL) significantly increased (**A**) transmucosal short circuit current (*I_sc_*), with a concomitant decrease in (**B**) epithelial potential difference (PD, apical-side negative), during pre-treatment of, and blockade of translocation of S. Typhimurium across *ex vivo* human ileal follicle-associated epithelium (FAE) mounted in Ussing chambers. (**C**) Trans-epithelial electrical resistance (TEER) was maintained throughout the experiment. N = 4, with 2 tissue replicates in each case. * *P*<0.05, ** *P*<0.01, ANOVA. For each tissue, *I*_sc_ and PD responses were calculated and expressed as the increment change (Δ) for each sampling period. Arrows at T_0_ min indicate addition of EGFP-expressing *S.* Typhimurium LT2 to the mucosal compartment (1×10^8^ CFU/mL). Overnight culture of Ussing chamber serosal medium following 2 h infection had already demonstrated soluble plantain fibre to block translocation of *Salmonella* across isolated human FAE in this experiment; see reference [21].

We have recently shown that plantain NSP (5 mg/mL) blocks translocation of EGFP-expressing *S.* Typhimurium LT2 across human ileal follicle-associated epithelium (FAE) mounted in Ussing chambers [21]. We now report that this is associated with a marked increase in transmucosal short circuit current (*I_sc_*) indicating increased ion efflux. Pre-treatment of tissue with 5 mg/mL plantain NSP for 20 min prior to infection, increased transmucosal *I_sc_*, with the peak change seen at T_30_ min post-infection (Δ*I_sc_* 5.86±1.89 µA.cm^−2^, N = 4, with n = 2 replicates; P<0.01 ANOVA vs. untreated control tissue (see **Figure 5A**). Concomitant changes in potential difference (PD) across ileal FAE were also observed in response to plantain NSP treatment; P<0.05 ANOVA (**Figure 5B**). *Salmonella* infection alone resulted in no change in transmucosal *I_sc_* nor electrical PD in non-plantain ileal FAE (**Figure 5A–B**). Trans-epithelial electrical resistance (TEER) was maintained throughout the 2 h Ussing chamber experiments of both infected plantain NSP pre-treated ileal FAE and buffer-treated controls, although interestingly there was a trend to a more stable TEER following treatment with soluble plantain fibre (**Figure 5C**).

### The inhibitory effect of plantain NSP on *Salmonella*-intestinal epithelial cell adherence is mediated primarily by the acid (pectic) polysaccharide fraction

**Figure 6:**
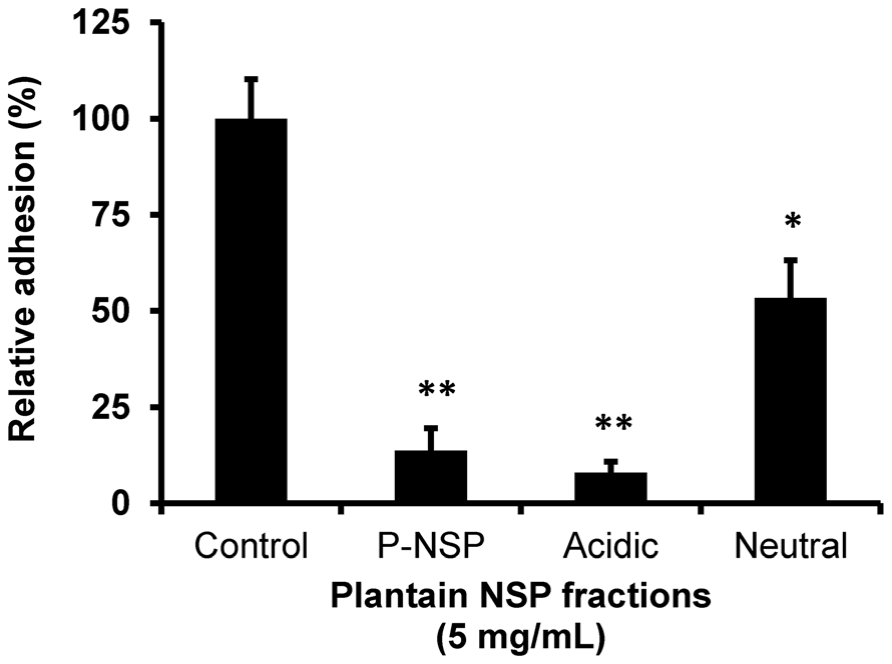
The inhibitory activity of soluble plantain NSP to block *Salmonella*-host intestinal epithelium interaction lies within an acidic polysaccharide component. At 5/mL, the acidic polysaccharide fraction of plantain NSP significantly blocked adhesion of *S*. Typhimurium LT2 to human intestinal Caco2 cells, whereas the neutral fraction had a lesser effect compared to vehicle-treated control (N = 2 experiments, n = 3 replicates; * *P*<0.05, ** *P*<0.01 Kruskal-Wallis).

At 5 mg/mL, the acidic polysaccharide fraction of plantain NSP isolated by Q-Sepharose® anion-exchange fractionation inhibited adhesion of *S*. Typhimurium LT2 to the human intestinal Caco2 cell-line by 92% (95% CI, 85–100) and was at least as effective as the unfractionated soluble plantain fibre 86% (95% CI, 85–100), whereas the neutral polysaccharide fraction had lower inhibitory activity (47% inhibition of adhesion (95% CI, 21–72); both *P*<0.05 compared to vehicle–treated control cells (N = 2 experiments, n = 3 replicates, Kruskal-Wallis; see **Figure 6**. The acidic fraction was also shown to inhibit adhesion of *S.* Typhimurium LT2 to porcine B1OXI enterocytes by 52% (95% CI, 27–76; *P*<0.05), with the neutral fraction having no inhibitory activity (N = 1, n = 4, *P*<0.01).

### Composition analysis reveals the pectic fraction of plantain NSP to be mainly or only homogalacturonan

The whole plantain NSP preparation as tested in these studies contained substantial maltodextrin (added up to 40% by weight to facilitate resolubilisation after freeze-drying – see Methods) and 2.5% by weight of galacturonic acid, indicating the presence of pectic material (**see File S3**). Composition of the acidic plantain NSP fraction, obtained by Q-Sepharose® anion-exchange fractionation contained approximately 15% (by weight total carbohydrate) of galacturonic acid. On acid hydrolysis and digestion with Driselase®, negligible rhamnose, galactose or arabinose was formed from the acidic fraction nor from the whole plantain NSP, indicating little or no rhamnogalacturonan-I and rhamnogalacturonan-II; **File S3**. Thus, the pectic material within the acidic fraction of plantain NSP is mainly or only homogalacturonan. The neutral plantain NSP fraction contained no detectable galacturonic acid indicating absence of pectic material.

## Discussion

These studies show that supplementation of pelleted feed with soluble plantain NSP was well tolerated by chickens and reduced *S. enterica* serovar. Typhimurium 4/74 translocation as shown by reduction in splenic bacteria. Histopathological findings were consistent with *S*. Typhimurium infection in birds of this age as previously described [22]. This is the first time that dietary supplementation with soluble plantain NSP has been shown to block bacterial invasion in an animal model. Soluble plantain NSP also reduced adherence of *S.* Typhimurium to caecal crypt epithelium in primary culture from both egg layers and broilers, and blocked adhesion and invasion of *S.* Typhimurium strains to porcine B1OXI enterocytes.

These results support previous studies from our group showing that soluble plantain NSP blocks adherence of colonic mucosa-associated adherent, invasive *E. coli* (AIEC) and *Salmonella* spp. to human intestinal cell-lines *in vitro*, with no evidence of any cytotoxicity to epithelial cell monolayers, nor any direct bactericidal activity [19]–[21]. Plantain NSP supplementation had no consistent effect on caecal lumen *Salmonella* counts. It is intriguing that soluble plantain fibre appears to exert its inhibitory effect on *Salmonella* translocation via action on the epithelium rather than through any interaction with bacterial carbohydrate-binding proteins (lectins/adhesins). Further studies using Ussing chamber experiments of human terminal ileum showed that soluble plantain NSP treatment of the epithelium increases basal transmucosal short circuit current (*I_sc_*), a measure of active (electrogenic) ion movements mediated by cellular transport processes [23]. The observed changes in Isc are likely as a result of increased epithelial chloride (Cl^−^) secretion, which as a physiological effect will cause efflux of ions and water transport, potentially impeding bacterial translocation. Electrogenic Cl^−^ secretion, and associated mucosal hydration, is now recognised as a key component of innate epithelial defence, influencing not only bacterial-epithelial interactions but also influencing the composition of the intestinal microbiome [24]. As part of these recent studies, secretagogue enhanced epithelial Cl^−^ secretion and water transport was shown to be particularly effective in reducing *Salmonella* internalisation to, and translocation across T84 colonocytes by over 70% [24]. Also of interest, is that addition of probiotics to broiler diets has showed similar increases in mid-jejunal and colonic mucosal *I_sc_*, attributed to increased sodium glucose transport [25].

*Salmonella* serovars such as *S*. Enteritidis and *S*. Typhimurium are thought to invade human host intestine initially via the microfold (M) cells of the follicle-associated epithelia (FAE) overlying Peyer's patches located in the distal ileum and colon [26], [27], with invasion of other epithelial cells only occurring after subsequent switch to expression of Spi2 (*Salmonella* pathogenicity island 2) encoding a type III secretory system to allow bacteria to enter intestinal cells basolaterally and/or directly by the apical pole [28]. *S.* Typhimurium has also been shown to transform epithelial cells of the FAE into M cells to enhance translocation across the intestinal mucosa [29]. M cells act to sample and translocate bacteria and antigens to mucosal lymphocytes, dendritic cells and macrophages located within basolateral pockets of the M cell. *S*. Typhimurium has also been reported to cross the epithelium via intra-epithelial dendritic cells [30]. FAE in the gastrointestinal tract of chickens also possess M-like cells that share the characteristic morphological and histochemical features of mammalian M cells [31]. The FAE structures in the chicken are much more diffuse than those seen in humans and perhaps not so developed with respect to antigen uptake [31], [32]. The large secondary lymphoid organs of the chicken gastrointestinal tract, the caecal tonsils, which are thought to be the primary site of bacterial invasion, also contain M-like cells [31], [33]. This is likely the main site of *Salmonella* invasion for chickens [6], [34]. Our own previous work has demonstrated that soluble plantain NSP can block translocation of AIEC, *Shigella* and *Salmonella* across *in vitro* modelled M cells, generated by co-culture of human Caco2 intestinal epithelial cells with Raji B lymphocytes [20]–[21]. We have also shown soluble plantain NSP to block translocation of AIEC and *S*. Typhimurium across *ex vivo* human ileal FAE mounted in Ussing chambers [20], [21].

The soluble plantain NSP inhibitory activity against bacteria-epithelial interaction is shown by our *in vitro* studies presented here to be largely due to the acidic polysaccharide or pectin fraction [35]. It was not feasible to generate the large amounts of this fraction needed to allow testing in the *in vivo* chick model but the relatively good inhibition achieved by the unfractionated plantain NSP implied that this would not be necessary nor indeed economic for large scale use.

Pectins comprise approximately one-third of the dry weight of primary cell walls (the predominant cell walls of the edible plant parts) in non-poalean monocots such as plantain [36], playing key roles in cell-wall structure and function [37], [38]. Pectins are complex polysaccharides, in which at least three distinct domains (homogalacturonan and the rhamnogalacturonans RG-I and RG-II) are covalently linked, the RGs containing large proportions of neutral sugars, especially l-rhamnose, d-galactose and l-arabinose, in addition to d-galacturonic acid [36]. Some pectic domains also carry methyl and acetyl ester groups [39]. Pectic polysaccharides interlink to each other, e.g. homogalacturonan domains via Ca^2+^ bridges [37] and RG-II domains via borate bridges [40]; and neutral wall polysaccharides such as xyloglucan can be glycosidically linked to RG-I [41]. Analysis in this study revealed that the acidic (pectic) fraction of plantain NSP possessing significant inhibitory activity against *Salmonella* adhesion and invasion was composed mainly or only of homogalacturonan, as negligible rhamnose, galactose or arabinose was formed following hydrolysis indicating absence of RG-I and RG-II. RG-II is resistant to Driselase, but would have been hydrolysed to monosaccharides by acid. The neutral plantain NSP fraction, which showed relatively little blockade of *Salmonella*-host cell interaction, contained no detectable pectic material. Pectins are not appreciably digested in the mammalian upper gut but are almost completely fermented in the large intestine [42]. Pectin from ginseng has been shown to possess anti-adhesive activity against other gut pathogens such as *Helicobacter pylori*, and some ability to inhibit haemagglutination by bacteria, including that caused by *Staphylococcus aureus* and *Propionibacterium acnes*, but not that effected by *E. coli* and *Lactobacillus acidophilus* [43].

Thus, soluble plantain NSP reduces adhesion and invasion of *Salmonella* spp. *in vitro*, in primary cell culture models, and *in vivo* in the chicken. This suggests that dietary supplementation with soluble plantain NSP has potential to achieve a useful protection against invasive salmonellosis in animals and man. The epithelial adhesion of other human pathogens such as enterotoxigenic *E. coli*, *Shigella sonnei*, and *Clostridium difficile* is also inhibited by soluble plantain NSP [20], [21]. We have recently described this action of dietary soluble NSP in inhibiting bacteria-host epithelium interactions as a ‘contrabiotic’ effect [44]. *In vitro* studies suggest that the acidic (homogalacturonan-rich) fraction is particularly inhibitory and the Ussing chamber studies of *ex vivo* human ileal cultures suggest that its effect is associated with a marked increase in epithelial ion secretion, most likely chloride. Further studies are indicated to assess the generalizability of this protective effect to other host and pathogen interactions *in vivo*.

## Materials and Methods

### Ethics statement

All work was conducted in accordance with UK legislation governing experimental animals under project licences PPL 40/3063 and PPL40/3652 and was approved by the University of Liverpool ethical review process prior to the award of the licence. Chicks were reared in the high-biosecurity poultry unit, University of Liverpool, in secure floor pens at a temperature of 30°C until 3 weeks of age, then at 20°C. Birds were allowed *ad libitum* access to water and vegetable protein-based laboratory poultry pelleted diets under test. All animals were checked a minimum of twice daily to ensure their health and welfare.

Studies described using human tissue specimens from macro- and microscopically normal terminal ileum were obtained from patients who underwent surgery for colon cancer and who had given their informed written consent as previously described [21]. The study was approved by the Regional Human Ethics Committee; Linköping, Sweden.

### Bacterial strains and growth conditions

*Salmonella enterica* serovar Typhimurium LT2 and *S*. Typhimurium 4/74 were obtained from Professor Craig Winstanley (Institute of Infection & Global Health, University of Liverpool) and Professor Mark Stevens (Roslin Institute, University of Edinburgh) respectively. *S*. Typhimurium 4/74 was used for *in vivo* chick studies, due to its high virulence [45]. Serovar *S.* Enteritidis (P125109) was obtained from Professor Paul Barrow (Veterinary Medicine, University of Nottingham). Bacteria were grown from frozen stocks on solid Luria-Bertani (LB) agar at 37°C, for 24 h. Prior to infection of cultured epithelial cells, all strains of Salmonellae were washed three times in sterile phosphate-buffered saline (PBS), pH 7.4 and re-suspended to an OD_600 nm_ of 1.0 (*S*. Typhimurium LT2 and *S.* Enteritidis) or 1.2 (*S.* Typhimurium 4/74), equating to ∼1×10^9^ CFU/mL.

*S.* Typhimurium LT2, transformed with plasmid pEGFP carrying the enhanced green fluorescent protein gene e*gfp*, was used in experiments examining bacterial translocation across *ex vivo* human follicle-associated epithelium (FAE) mounted in Ussing chambers, as previously described [21].

### Soluble plantain fibre (non-starch polysaccharide) preparation

Non-starch polysaccharide (NSP) preparations from Confoco plantain flour (Trobana Green Plantain flour; Confoco International Ltd; Ripley, UK) were prepared by Provexis Plc (Windsor, UK) at the Teagasc Food Research Centre (Moorepark, Ireland). In brief, dry plantain flour was homogenised in reverse-osmosis purified water (ratio 1∶2), heated to between 90°C and 100°C for 10 min with continuous high-shear mixing to effect starch swelling and gelatinisation. Following cooling to 25°C, the homogenate was treated with fungal α-amylase Fungamyl® (Novozymes; Bagsvaerd, Denmark) for 2 h at pH 6–7. The mixture was then heated to 72°C for 20 min to fully inactive the Fungamyl enzyme. Insoluble NSP was removed by centrifugation and subsequently, low molecular weight components (<300 Da), including starch degradation products, were removed from the soluble NSP by nanofiltration. The concentrated retentate (containing in addition up to 45% by weight plantain-derived maltodextrin carrier as part of the bulk manufacturing process to counter difficulties in freeze drying/resolubilisation) was spray-dried to a fine dry powder with a particle size distribution of 50–100 µm and a bulk density of 175 g/L (see **File S4**).

Plantain NSP concentrations tested for the *in vitro* studies were selected to be within the range of effective luminal concentrations in the human distal colon that would be readily achievable with dietary supplementation [20] (around 5 mg/mL, observed to inhibit adhesion of adherent, invasive *E. coli*, *Salmonella* and *Shigella* to human intestinal epithelial cell-lines [19]–[21]. For the *in vivo* study, given that chickens each have two caeca with a typical volume of about 1 mL each, usually emptied twice per day, a minimum dietary intake of soluble plantain fibre to give a maximum inhibitory effect on bacterial adhesion (achieved with a final concentration of 5 mg/mL [21]) was calculated to be ∼20 mg/d. A typical chick feed intake is 20 g/d of which 5% (i.e. 1 g/d) would usually be fibre. Plantain NSP supplementation was therefore evaluated in the range of 0–200 mg/d/chick.

### Preparation of purified acidic and neutral polysaccharide fractions from soluble plantain NSP

Initial analytical fractionation of soluble plantain NSP (1.6 g dissolved in 50 mL 50 mM Tris-HCl, pH 7.4) using a HiPrep™ Q-Sepharose® FF 16/10 anion-exchange column on an ÄKTAprime plus liquid chromatography system (GE Healthcare Life Sciences, Chalfont St Giles, UK) demonstrated that bound acidic polysaccharides (as determined by uronic acid content) could be eluted step-wise in 50 mM Tris-HCl buffer containing 0.1, 0.5 and 1 M NaCl at a flow rate of 5 mL/min (data not shown). Using this information, a bulk preparation of both neutral and acidic fractions of soluble plantain NSP was then conducted using preparative Q-Sepharose® (counter-ion Cl^−^) Fast Flow anion-exchange medium (GE Healthcare) in a 2.5 litre container [46]. Q-Sepharose (300 mL) was washed extensively with three 1 L volumes of sterile deionised water and then equilibrated twice with 1 L of sterile-filtered 50 mM Tris-HCl buffer, pH 7.4. Plantain NSP (25 g) was added to 750 mL sterile 50 mM Tris-HCl, pH 7.4, mixed thoroughly for 1 h at room temperature and left to settle overnight at 4°C. The majority upper, clear layer (∼700 mL) was removed, filtered under vacuum through a sintered glass funnel and Whatman No.1 filter paper, and then added to a 2.5 L mixing vessel containing Q-Sepharose® and rotated for 1 h, at 4°C. Unbound neutral polysaccharide was collected and filtered again. Following two 15 min washes with equilibration buffer to remove any residual unbound material, Q-Sepharose®-bound acidic polysaccharides were eluted with 800 mL 1 M NaCl in 50 mM Tris-HCl, with rotating overnight at 4°C.

Neutral and acidic polysaccharide fractions were then desalted using multiple pre-packed PD MidiTrap G-10 gravity mini-columns (1 mL per column), eluted with sterile deionised water as per the manufacturer's instructions (GE Healthcare). Elution profiles for the purified neutral and acidic plantain polysaccharides on the G-10 mini-columns were established (**File S5**). Fractions were assayed for total carbohydrate content, and the void fraction (approximate M_r_ >700) was collected. All columns were calibrated using phenol red (354 Da) as a low molecular size marker. Desalted fractions were shell-frozen in round-bottomed glass vacuum flasks by immersion and rapid rotation in 100% ethanol containing dry ice. Flasks were stored for a least 20 min at −80°C before lyophilisation overnight under vacuum. The total yield of acidic material from 25 g plantain NSP was 1.16 g (4.64% by weight); the total yield of neutral material was 4.21 g (16.84% by weight).

### Assessment of total carbohydrate and uronic acid content of chromatography fractions

Total carbohydrate content of NSP fractions was assayed using a modified method of Dubois *et al.* [47]. Briefly, 10 µL fractions were added to 96-well microtitre plates (Corning/Costar) in triplicate, and 100 µL of 4% (wt/vol) phenol dissolved in deionised water was added at room temperature for 5 min. Concentrated sulphuric acid (150 µL) was then rapidly delivered to all wells (with great care) and vigorously aspirated to generate heat required for reaction colour development. Plates were left to cool for 20 min and then measured for *A*_560_. Carbohydrate content of samples was determined using a calibration curve of d-glucose (0–20 µg/mL). Hexuronic acid content (d-glucuronic acid and d-galacturonic acid) was measured using a commercial K-URONIC assay (Megazyme International; Bray, Ireland). Increase in absorbance at 340 nm was determined upon incubation of fractions or 0–150 µg of d-glucuronic acid with uronate dehydrogenase in the presence of nicotinamide adenine dinucleotide (NAD^+^) at 25°C for 10 min, as per manufacturer's instructions.

### Analysis of the hydrolysis products of plantain NSP, and the neutral and acidic NSP anion-exchange fractions

Plantain NSP and Q-Sepharose derived neutral and acidic NSP fractions were each hydrolysed with either 0.5% Driselase® (a commercial enzyme mixture of hydrolytic enzymes capable of digesting homogalacturonan and rhamnogalacturonan-I (RG-I) efficiently to galacturonic acid and associated neutral monosaccharides) or 2 M trifluoroacetic acid (TFA) as per [48]. Thin-layer chromatography (TLC) was performed on Merck silica-gel plates and on plates pre-washed for in acidified acetone to enhance mobility of the uronic acids. Each loading was derived from 25 µg of plantain NSP or polysaccharide fraction (or contained an equivalent amount of Driselase® or TFA). Plates were run under two solvent conditions, ethyl acetate/pyridine/acetic acid/water (6∶3∶1∶1) and butan-1-ol/acetic acid/water (2∶1∶1), each followed by staining using thymol/H_2_SO_4_. To better determine galacturonic acid yields, high-voltage paper electrophoresis (HVPE) of plantain fibre samples and their hydrolysis products was also performed as per [49]. Briefly, each loading was derived from 200 µg of the fibre or fraction (or contained an equivalent amount of Driselase® or TFA). Electrophoresis was performed using Whatman No. 1 paper in pH 2.0 buffer at 4.7 kV for 80 min, with monosaccharide and oligogalacturonide markers included for reference. Staining was with aniline hydrogen-phthalate [49].

### *Salmonella* adhesion and invasion assays in mammalian and avian epithelial cells

The enterocyte-like B1OXI cell-line (BioNutriTech; Montpellier, France) originally thought to be from dissected colonic tissue of 19-day old chicken embryos [50], but recently verified as porcine in origin [51] was seeded at 1×10^6^ cells/well in 24-well tissue culture plates (Costar; High Wycombe, UK) and maintained in advanced Dulbecco's-modified Eagle's medium (DMEM), supplemented with 5% (vol/vol) fetal calf serum (FCS) (Invitrogen; Paisley, Scotland). Media was supplemented with 100 U/mL penicillin, 100 µg/mL streptomycin and 8 mM glutamine (Sigma-Aldrich; Poole, UK). Cultures were maintained at 37°C in a humidified atmosphere of 5% (vol/vol) CO_2_, 95% air for 24 h

Prior to infection with *Salmonella* strains, confluent cells were washed three times with sterile PBS and cultured overnight in DMEM without antibiotics. Following 30 min pre-treatment of cells with or without soluble plantain NSP (0 to 10 mg/mL in antibiotic-free DMEM), cells were infected at a multiplicity of infection (MOI) of 20, for 90 min. Each monolayer was then washed with sterile PBS to remove non-adherent bacteria and adherence to, and invasion of, epithelial cells assessed by gentamicin protection assay, as per [19], [21]. Cells were lysed with sterile 1% (vol/vol) Triton X-100, serial dilutions performed and bacteria enumerated in triplicate, following overnight growth on LB agar.

Additional experiments were also performed to determine whether action of plantain NSP to block *Salmonella* adhesion was via an action on the epithelial monolayer or direct interaction with bacteria. To test the former, plantain NSP was added to B1OXI cells 30 min prior to infection as described above , but then removed by three washes with sterile PBS (1 min each; at 37°C). Monolayers were then provided fresh antibiotic-free DMEM, infected and levels of adherent *Salmonella* assessed. To test for direct interaction with bacteria, plantain NSP was pre-incubated with *Salmonella* for 30 min, followed by centrifugation, re-suspension of bacteria in anti-biotic free media and inoculation of epithelial cell monolayers.

Plantain NSP blockade of *S.* Typhimurium 4/74 was also examined *in vitro* using the human colorectal adenocarcinoma cell-line Caco2, as this isolate was to used in the *in vivo* studies, and to compare to our previous studies using *S*. Typhimurium LT2 [21].

As per previous studies [20], [21], epithelial cell viability during plantain NSP treatment and infection was carefully monitored by measurement of adenylate kinase released to the culture medium using a ToxiLight™ bioassay kit (Lonza; Walkersville, USA), and confirmed in selected experiments by Giemsa microscopy.

### Primary chick caecal crypt culture and *Salmonella* infection

Crypts were isolated from the caeca of 14-day old Lohmann Brown Classic egg-layer chickens and, in separate experiments, from the caeca of 33-day old Hubbard JA57 broiler chickens using methods adapted from Van Deun *et al* [52]. In brief, caeca were transported on ice and washed in Hank's balanced salt solution (HBSS) supplemented with 20 mM HEPES, 100 U/mL penicillin, 100 µg/mL Streptomycin, 50 µg/mL gentamicin and 2 mM L-glutamine. Tissue was cut into smaller pieces and digested in supplemented HBSS containing 2.5% (vol/vol) FCS, 40 µg/mL dispase (Roche; Little Chalfont, UK) and 150 U/mL collagenase XI (Sigma), in a shaking water bath (120 rpm) at 37°C for 50 min. Crypts were then passed through a 200 µm nylon filter membrane, and collected in 40 µm filters. Crypts were subsequently plated into 6-well tissue culture plates coated with bovine Type I collagen (Inamed BioMaterials; Fremont, USA) and maintained in DMEM containing 2% (vol/vol) FCS, supplemented with 6% (vol/vol) chicken serum, 100 U/mL penicillin, 100 µg/mL streptomycin, 2 mM L-glutamine, 20 mM HEPES, 10 µg/mL bovine insulin, 1.4 µg/mL hydrocortisone, 1 µg/mL fibronectin, 5 µg/mL transferrin and 50 µg/mL gentamicin; all supplements were from Sigma. Crypts were incubated at 37°C in 5% CO_2_, 95% air, and washed daily with sterile PBS, and media was replaced with supplemented media but with reduced FCS and chicken serum (both 0.5% vol/vol). Crypt viability was determined by measurement of adenylate kinase released to the culture medium using a ToxiLight™ bioassay kit (Lonza).

Infection assays were all performed 4 d following initial isolation of caecal crypts (seeded at ∼7×10^3^ crypts per well), with crypts (absent of any contaminating fibroblasts) pre-treated for 30 min either with soluble plantain NSP (10 mg/mL, in DMEM without antibiotics) or vehicle. *S*. Typhimurium 4/74 (4.2×10^7^ bacteria) were added to each well, prior to a 90 min incubation. Crypts were lysed with sterile 1% (vol/vol) Triton-X, serial dilutions performed and adherent bacteria enumerated following overnight growth on Brilliant Green agar (Oxoid; Basingstoke, UK).

### Confirmation of bacterial invasion by microscopy

Epithelial cells were seeded at 3×10^6^ cells/mL onto 13 mm glass cover slips inside 6-well tissue culture plate wells (Costar Corning) and were incubated for 24 h at 37°C. Cells were washed three times with sterile PBS before pre-treatment for 30 min with or without plantain NSP (10 mg/mL, in DMEM without antibiotics). Cells were infected with bacteria (MOI of 60) for 150 min at 37°C, followed by five washes with sterile PBS before being fixed with 70% (vol/vol) ethanol in sterile water, for 20 min at room temperature. Cells were washed a further three times with sterile PBS prior to staining with 10% Giemsa solution (Sigma) for 30 min at room temperature. Cells were then further washed with sterile water and coverslips mounted onto glass microscope slides using distyrene plasticizer and xylene (DPX). Images were taken using a Hitachi HV-C20A microscope camera (version 5.0.2 software) on a Leica AS/LMD microscope system.

### Examination of epithelium electrophysiological factors following plantain blockade of *Salmonella* translocation across isolated human ileal FAE

Uptake studies of enhanced green fluorescent protein (EGFP)-expressing *S.* Typhimurium LT2 across FAE from macro- and microscopically normal human terminal ileum, with pre-incubation for 20 min with either 5 mg/mL plantain NSP or Kreb's buffer vehicle, were previously performed in Ussing chambers as per [21]. Transmucosal electrophysiological parameters of tissue (*I_sc_*, PD and TEER) were monitored throughout.

### Assessment of the effect of chick feed supplementation with plantain NSP on *Salmonella* infection *in vivo*

Inbred specified pathogen-free White Leghorn Line 0 chicks were obtained on day of hatch from the Pirbright Institute (Compton Laboratory; Newbury, UK), divided into treatments groups and housed in secure floor pens at a temperature of 30°C, with water available *ad libitum*. Birds were fed custom-made commercial vegetable protein-based pellet diets (with a fibre content of ∼5% but free of soluble fibre) with or without supplementation with soluble plantain NSP (SDS; Witham, UK). Diets supplemented with plantain NSP were at levels equivalent to a daily intake of 12.5, 50 and 200 mg NSP per bird. At 8 d of age, chicks were inoculated by gavage with 4×10^8^ *S*. Typhimurium 4/74. Subsequently, birds from each group were sacrificed 3, 6 and 10 d post-infection. Liver, spleen, ileum, caeca and caecal contents were sequentially removed from each bird. Splenic and liver tissue were homogenised diluted 1∶10 (wt/vol) in sterile PBS in a Colworth 80 stomacher (AJ Seward & Co. Ltd.; London, UK), whilst caecal contents were vortexed in PBS, and then all samples were serially diluted and plated onto Brilliant Green agar (Oxoid) to enumerate *S*. Typhimurium (pink/red) from other bacteria (green/yellow). Any samples yielding negative CFU were re-grown overnight at 37°C in Selenite F broth (Oxoid) at a ratio of 1∶1. Broth was then re-plated to Brilliant green agar overnight to confirm absence of *S*. Typhimurium.

### Avian histopathology

Post-mortem organs (spleen, liver, caeca and ileum) were each fixed in 4% (wt/vol) paraformaldehyde and embedded in paraffin wax. Sections (3–5 µm) were prepared, dewaxed, rehydrated stepwise through ethanol to sterile water and then stained with hematoxylin and eosin. Tissue sections were reviewed by a qualified veterinary pathologist who was blind to the treatment groups.

### Statistical analyses

For the *in vitro* studies, N numbers indicate the total number of independent experiments performed, where each experiment was performed using n replicates for any individual treatment group. For the *in vivo* studies, N = number of birds in each treatment group, sacrificed on each day post-infection, with duplicates performed for all sample analyses. Independent treatment groups were assessed for normality and equality of variances, before analysis using Mann-Whitney U test, one-way analysis of variance (ANOVA) followed by selected pairwise comparisons (Bonferroni test) or non-parametric Kruskal-Wallis ANOVA followed by all pairwise comparisons (Conover-Inman) as appropriate. Data are presented as mean ± standard error of the mean (SEM). Differences were considered significant when *P*<0.05.

## Supporting Information

File S1 **Contains: Figure S1: Soluble plantain NSP inhibits adhesion of** ***S*** **. Typhimurium strains LT2 and 4/74 to the human intestinal Caco2 cell-line** ***in vitro*** **.** Pre-treatment (30 min) with soluble plantain NSP dose-dependently blocked (**A**) adhesion and (**B**) invasion of *S*. Typhimurium 4/74 to human Caco2 cells, at similar levels to that observed for *S*. Typhimurium LT2 (N = 3 experiments, n = 4 replicates; *P<0.05, ** *P*<0.01, *** *P*<0.001, Kruskal-Wallis). Data (mean ± SEM) expressed relative to adherence (or invasion) of vehicle-treated control (100%).
(PDF)

File S2 **Contains: Figure S2: Soluble plantain NSP blocks adhesion of** ***S*** **. Enteritidis to the porcine enterocyte cell-line B1OXI** ***in vitro*** **.** Pre-treatment with soluble plantain NSP at 10 mg/mL blocked (**A**) adhesion to, and (**B**) invasion of *S*. Enteritidis to B1OXI cells (N = 3, n = 4; ***P<0.001 Mann Whitney U). Data (mean ± SEM) expressed relative to adherence (or invasion) of vehicle-treated control (100%).
(PDF)

File S3 **Contains: Figure S3A: Thin-layer chromatography (TLC) of whole plantain NSP, preparative Q-Sepharose neutral and acidic polysaccharide fractions and their hydrolysis products.** Samples were hydrolysed with either trifluoroacetic acid (TFA) or Driselase. Each loading was derived from 25 µg of plantain NSP or polysaccharide fraction (the acidic fraction subjected to TLC had been reconstituted in physiological saline and contained ∼70% by weight salt, and thus its loading was ∼7.5 µg carbohydrate; blanks contained an equivalent amount of Driselase or TFA). All samples contained in addition up to 45% plantain-derived maltodextrin carrier (responsible for the glucose content). Samples were loaded on to Merck silica-gel plates pre-washed in acidified acetone to enhance the mobility of the uronic acids. The running solvent was ethyl acetate/pyridine/acetic acid/water (6∶3∶1∶1). The stain was thymol/H_2_SO_4_. **Figure S3B: High-voltage paper electrophoresis (HVPE) of whole plantain NSP, preparative Q-Sepharose neutral and acidic polysaccharide fractions and their hydrolysis products.** Samples were hydrolysed with either trifluoroacetic acid (TFA) or Driselase. Each loading was derived from 200 µg of plantain NSP or polysaccharide fraction (the acidic fraction subjected to HVPE had been reconstituted in physiological saline and was ∼70% by weight salt, thus its loading was ∼60 µg carbohydrate; blanks contained an equivalent amount of Driselase or TFA). As before all samples contained in addition up to 45% plantain-derived maltodextrin. Electrophoresis was performed on Whatman No. 1 paper in pH 2.0 buffer at 4.7 kV for 80 min. Staining was with aniline hydrogen-phthalate.
(PDF)

File S4 **Contains: Table S4:** Water soluble non-starch polysaccharide preparation derived from plantain (*Musa* AAB (Horn)), in powder format, containing in addition up to 45% plantain-derived maltodextrin carrier, and nature-equivalent colours and flavours.
(PDF)

File S5 **Contains: Figure S5: Desalting of Q-Sepharose anion-exchange chromatography fractions from soluble plantain NSP.** Following preparative anion-exchange chromatography, sodium chloride-eluted acidic polysaccharides (**A**) and unbound neutral polysaccharide (**B**) fractions were desalted into water using multiple PD MidiTrap™ G-10 gravity columns. Carbohydrate content of eluted fractions was measured by the phenol-sulphuric acid assay (blue line). Columns were pre-calibrated with the low molecular size marker phenol red (354 Da), measured as *A*_560_ (red line). Arrows indicate totally included (Vt) column volume. The solid bar indicates the elution fractions collected for lyophilisation and bioassay.
(PDF)
