# Supplementary material for: Dietary Supplementation with Soluble Plantain Non-Starch Polysaccharides Inhibits Intestinal Invasion of Salmonella Typhimurium in the Chicken
Source: PLoS One. 2014 Feb 3;9(2):e87658. doi: 10.1371/journal.pone.0087658 (PMC3911995; doi:10.1371/journal.pone.0087658)

Dietary supplementation with soluble plantain non-starch polysaccharides inhibits intestinal invasion of Salmonella Typhimurium in the chicken. Bryony N. Parsons, Paul Wigley, Hannah L. Simpson, Jonathan M. Williams, Suzie Humphrey, Anne-Marie Salisbury, Alastair J. M. Watson, Stephen C. Fry, David O'Brien, Carol L Roberts, Niamh O'Kennedy, Åsa V. Keita, Johan D. Söderholm, Jonathan M. Rhodes and Barry J. Campbell.

Supporting Information File S1:

Figure S1: Soluble plantain NSP inhibits adhesion of *S*. Typhimurium strains LT2 and 4/74 to the human intestinal Caco2 cell-line *in vitro*. Pre-treatment (30 min) with soluble plantain NSP dose-dependently blocked **(A)** adhesion and **(B)** invasion of *S*. Typhimurium 4/74 to human Caco2 cells, at similar levels to that observed for *S*. Typhimurium LT2 (N=3 experiments, n=4 replicates; **P*<0.05, ** *P*<0.01, *** *P*<0.001, Kruskal-Wallis). Data (mean ± SEM) expressed relative to adherence (or invasion) of vehicle-treated control (100%).

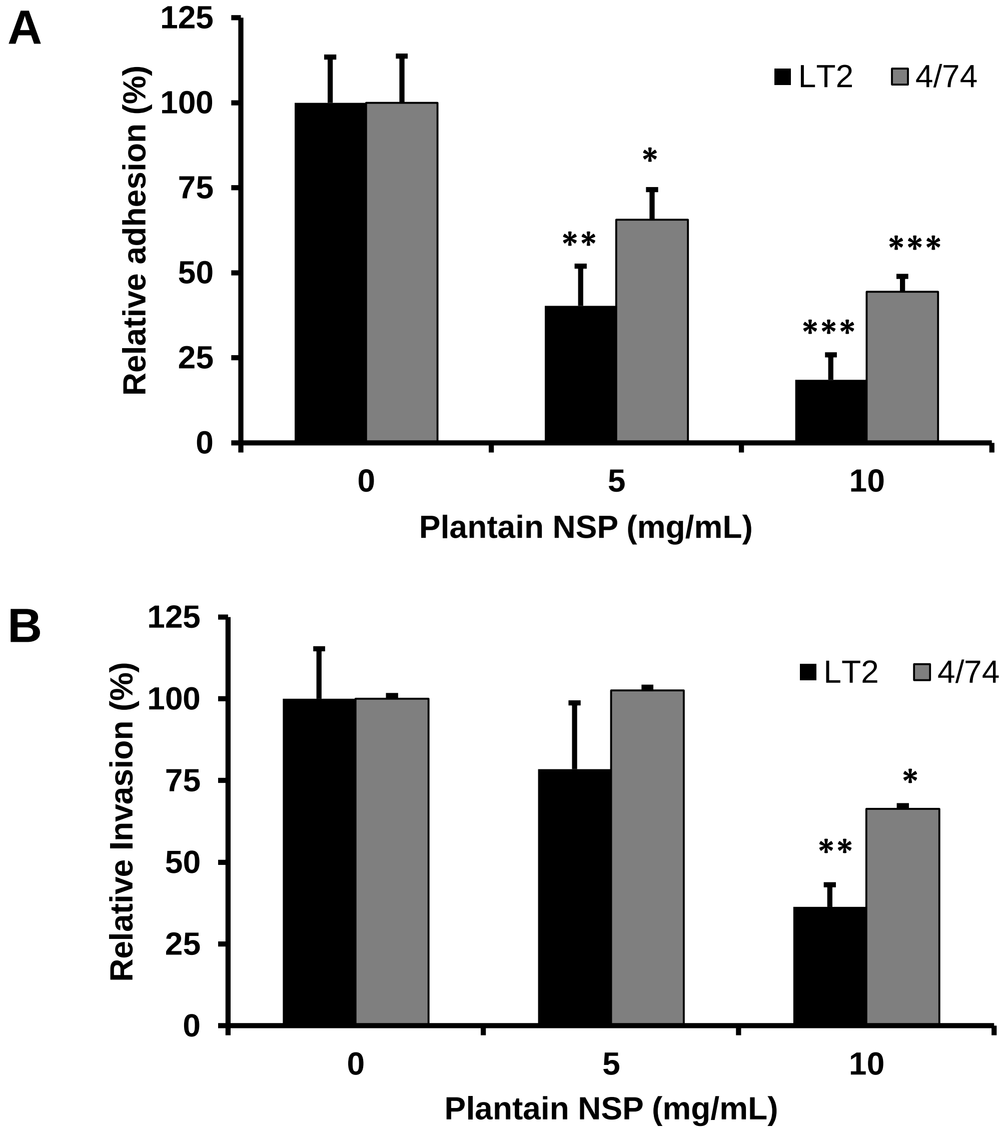

Supplement: File S1 — Contains: Figure S1: Soluble plantain NSP inhibits adhesion of S . Typhimurium strains LT2 and 4/74 to the human intestinal Caco2 cell-line in vitro . Pre-treatment (30 min) with soluble plantain NSP dose-dependently blocked (A) adhesion and (B) invasion of S. Typhimurium 4/74 to human Caco2 cells, at similar levels to that observed for S. Typhimurium LT2 (N = 3 experiments, n = 4 replicates; *P<0.05, ** P<0.01, *** P<0.001, Kruskal-Wallis). Data (mean ± SEM) expressed relative to adherence (or invasion) of vehicle-treated control (100%). (PDF) [file pone.0087658.s001.pdf]
