# Supplementary material for: Dietary Supplementation with Soluble Plantain Non-Starch Polysaccharides Inhibits Intestinal Invasion of Salmonella Typhimurium in the Chicken
Source: PLoS One. 2014 Feb 3;9(2):e87658. doi: 10.1371/journal.pone.0087658 (PMC3911995; doi:10.1371/journal.pone.0087658)

Dietary supplementation with soluble plantain non-starch polysaccharides inhibits intestinal invasion of Salmonella Typhimurium in the chicken. Bryony N. Parsons, Paul Wigley, Hannah L. Simpson, Jonathan M. Williams, Suzie Humphrey, Anne-Marie Salisbury, Alastair J. M. Watson, Stephen C. Fry, David O'Brien, Carol L Roberts, Niamh O'Kennedy, Åsa V. Keita, Johan D. Söderholm, Jonathan M. Rhodes and Barry J. Campbell.

Supporting Information file S2

Figure S2: Soluble plantain NSP blocks adhesion of *S*. Enteritidis to the porcine enterocyte cell-line B1OXI *in vitro*. Pre-treatment with soluble plantain NSP at 10 mg/mL blocked **(A)** adhesion to, and **(B)** invasion of *S*. Enteritidis to B1OXI cells (N=3, n=4; ***P<0.001 Mann Whitney U). Data (mean ± SEM) expressed relative to adherence (or invasion) of vehicle-treated control (100%).

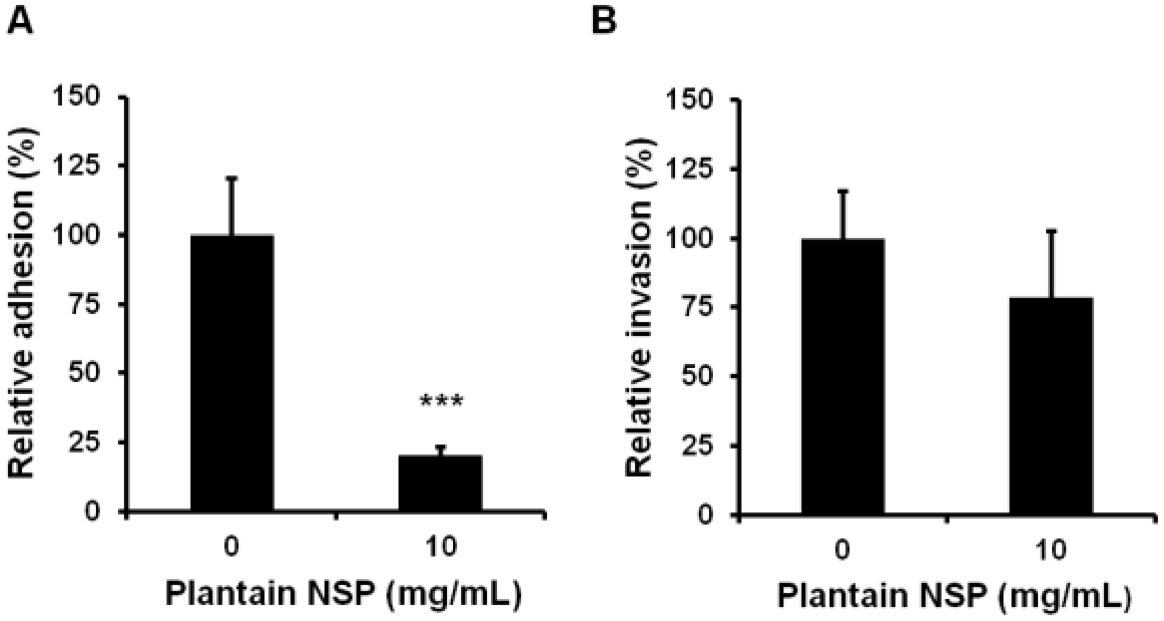

Supplement: File S2 — Contains: Figure S2: Soluble plantain NSP blocks adhesion of S . Enteritidis to the porcine enterocyte cell-line B1OXI in vitro . Pre-treatment with soluble plantain NSP at 10 mg/mL blocked (A) adhesion to, and (B) invasion of S. Enteritidis to B1OXI cells (N = 3, n = 4; ***P<0.001 Mann Whitney U). Data (mean ± SEM) expressed relative to adherence (or invasion) of vehicle-treated control (100%). (PDF) [file pone.0087658.s002.pdf]
