# Supplementary material for: Dietary Supplementation with Soluble Plantain Non-Starch Polysaccharides Inhibits Intestinal Invasion of Salmonella Typhimurium in the Chicken
Source: PLoS One. 2014 Feb 3;9(2):e87658. doi: 10.1371/journal.pone.0087658 (PMC3911995; doi:10.1371/journal.pone.0087658)
Supplement: File S3 — Contains: Figure S3A: Thin-layer chromatography (TLC) of whole plantain NSP, preparative Q-Sepharose neutral and acidic polysaccharide fractions and their hydrolysis products. Samples were hydrolysed with either trifluoroacetic acid (TFA) or Driselase. Each loading was derived from 25 µg of plantain NSP or polysaccharide fraction (the acidic fraction subjected to TLC had been reconstituted in physiological saline and contained ∼70% by weight salt, and thus its loading was ∼7.5 µg carbohydrate; blanks contained an equivalent amount of Driselase or TFA). All samples contained in addition up to 45% plantain-derived maltodextrin carrier (responsible for the glucose content). Samples were loaded on to Merck silica-gel plates pre-washed in acidified acetone to enhance the mobility of the uronic acids. The running solvent was ethyl acetate/pyridine/acetic acid/water (6∶3∶1∶1). The stain was thymol/H2SO4. Figure S3B: High-voltage paper electrophoresis (HVPE) of whole plantain NSP, preparative Q-Sepharose neutral and acidic polysaccharide fractions and their hydrolysis products. Samples were hydrolysed with either trifluoroacetic acid (TFA) or Driselase. Each loading was derived from 200 µg of plantain NSP or polysaccharide fraction (the acidic fraction subjected to HVPE had been reconstituted in physiological saline and was ∼70% by weight salt, thus its loading was ∼60 µg carbohydrate; blanks contained an equivalent amount of Driselase or TFA). As before all samples contained in addition up to 45% plantain-derived maltodextrin. Electrophoresis was performed on Whatman No. 1 paper in pH 2.0 buffer at 4.7 kV for 80 min. Staining was with aniline hydrogen-phthalate. (PDF) [file pone.0087658.s003.pdf]

Dietary supplementation with soluble plantain non-starch polysaccharides inhibits intestinal invasion of Salmonella Typhimurium in the chicken. Bryony N. Parsons, Paul Wigley, Hannah L. Simpson, Jonathan M. Williams, Suzie Humphrey, Anne-Marie Salisbury, Alastair J. M. Watson, Stephen C. Fry, David O'Brien, Carol L Roberts, Niamh O'Kennedy, Åsa V. Keita, Johan D. Söderholm, Jonathan M. Rhodes and Barry J. Campbell.

Supporting Information File S3:

Figure S3A: Thin-layer chromatography (TLC) of whole plantain NSP, preparative Q-Sepharose neutral and acidic polysaccharide fractions and their hydrolysis products. Samples were hydrolysed with either trifluoroacetic acid (TFA) or Driselase. Each loading was derived from 25 µg of plantain NSP or polysaccharide fraction (the acidic fraction subjected to TLC had been reconstituted in physiological saline and contained ~70% by weight salt, and thus its loading was ~7.5 µg carbohydrate; blanks contained an equivalent amount of Driselase or TFA). All samples contained in addition up to 45% plantain-derived maltodextrin carrier (responsible for the glucose content). Samples were loaded on to Merck silica-gel plates pre-washed in acidified acetone to enhance the mobility of the uronic acids. The running solvent was ethyl acetate/pyridine/acetic acid/water (6:3:1:1). The stain was thymol/H_2SO_4.

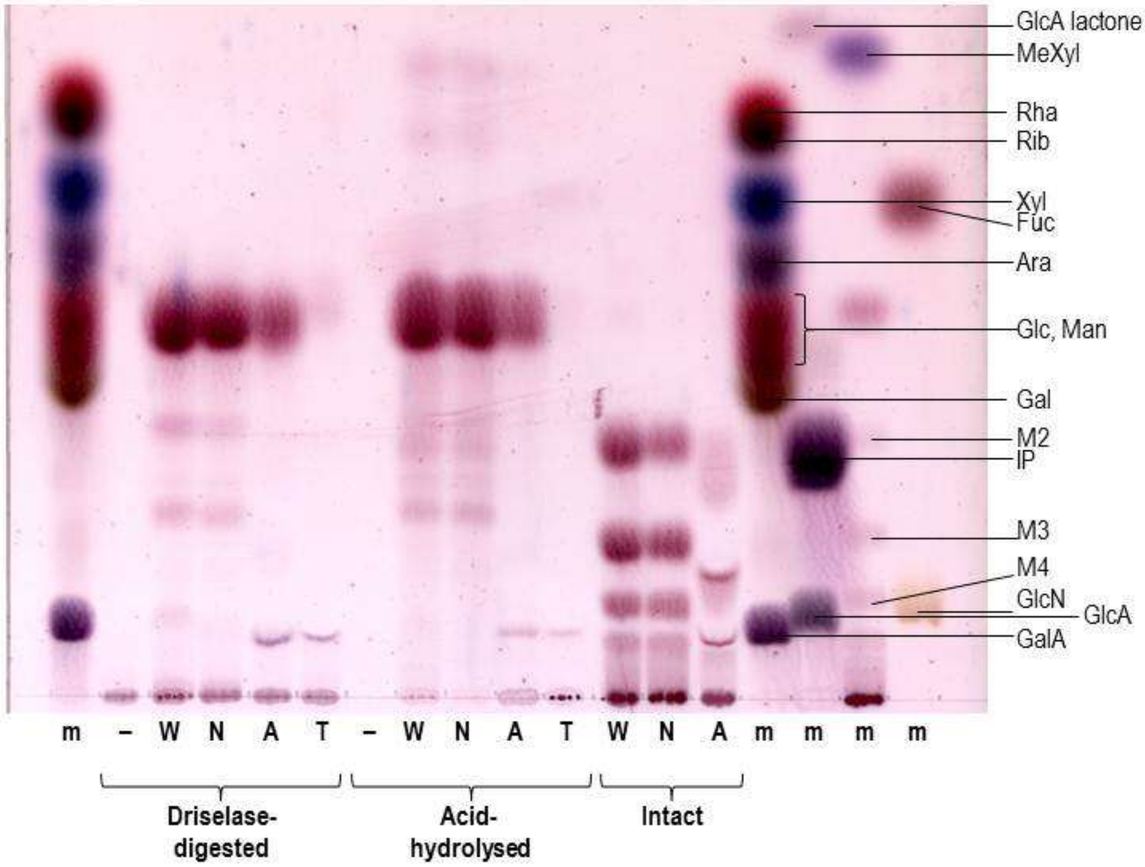

W, whole plantain NSP; N, neutral fraction; A, acidic fraction of plantain fibre; T, tomato hemicellulose; – , control without fibre; m, marker lanes [Fuc, fucose; GalA, galacturonic acid; Glc, glucose; GlcA, glucuronic acid; GlcN, glucosamine; IP, isoprimeverose; M2, maltose; M3–M4, maltotriose etc.; Man, mannose; Rha, rhamnose; Rib, ribose; Xyl, xylose].

Dietary supplementation with soluble plantain non-starch polysaccharides inhibits intestinal invasion of Salmonella Typhimurium in the chicken. Bryony N. Parsons, Paul Wigley, Hannah L. Simpson, Jonathan M. Williams, Suzie Humphrey, Anne-Marie Salisbury, Alastair J. M. Watson, Stephen C. Fry, David O'Brien, Carol L Roberts, Niamh O'Kennedy, Åsa V. Keita, Johan D. Söderholm, Jonathan M. Rhodes and Barry J. Campbell.

Figure S3B: High-voltage paper electrophoresis (HVPE) of whole plantain NSP, preparative Q-Sepharose neutral and acidic polysaccharide fractions and their hydrolysis products. Samples were hydrolysed with either trifluoroacetic acid (TFA) or Driselase. Each loading was derived from 200 µg of plantain NSP or polysaccharide fraction (the acidic fraction subjected to HVPE had been reconstituted in physiological saline and was ~70% by weight salt, thus its loading was ~60 µg carbohydrate; blanks contained an equivalent amount of Driselase or TFA). As before all samples contained in addition up to 45% plantain-derived maltodextrin. Electrophoresis was performed on Whatman No. 1 paper in pH 2.0 buffer at 4.7 kV for 80 min. Staining was with aniline hydrogen-phthalate.

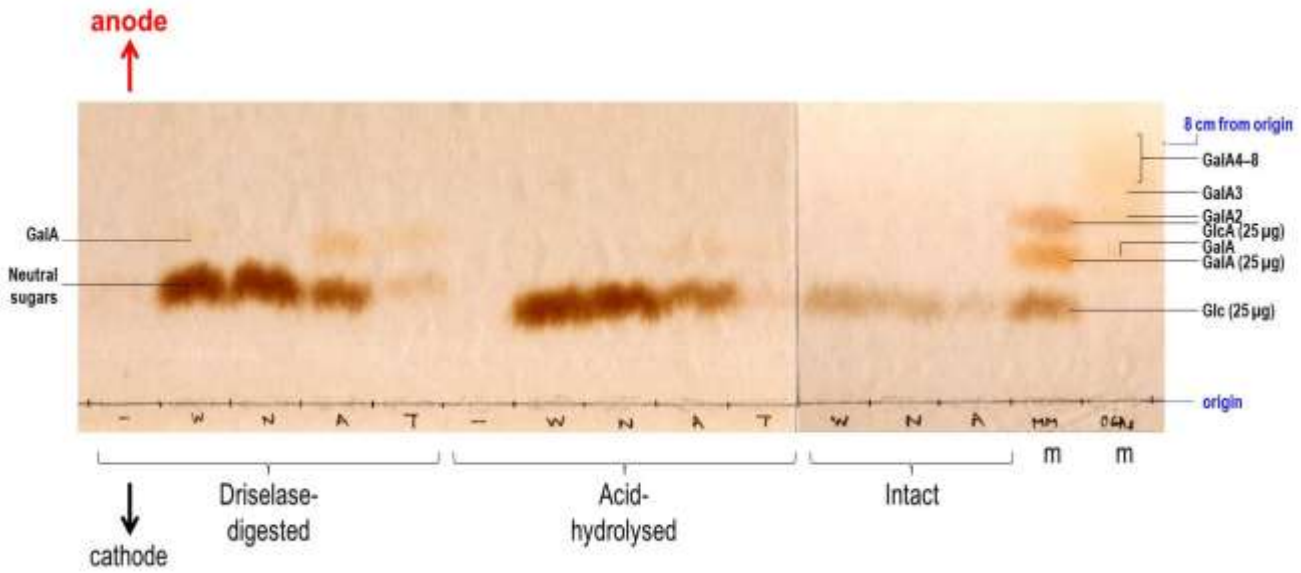

W, whole plantain NSP; N, neutral fraction; A, acidic fraction of plantain fibre; T, tomato hemicellulose; – , control without fibre; m, marker lanes [MM, markers - 25 µg each GalA, galacturonic acid; Glc, glucose; GlcA, glucuronic acid; and OGAs, oligogalacturonide markers of degree of polymerisation 1–8].
