# Supplementary material for: Dietary Supplementation with Soluble Plantain Non-Starch Polysaccharides Inhibits Intestinal Invasion of Salmonella Typhimurium in the Chicken
Source: PLoS One. 2014 Feb 3;9(2):e87658. doi: 10.1371/journal.pone.0087658 (PMC3911995; doi:10.1371/journal.pone.0087658)
Supplement: File S4 — Contains: Table S4: Water soluble non-starch polysaccharide preparation derived from plantain (Musa AAB (Horn)), in powder format, containing in addition up to 45% plantain-derived maltodextrin carrier, and nature-equivalent colours and flavours. (PDF) [file pone.0087658.s004.pdf]

Dietary supplementation with soluble plantain non-starch polysaccharides inhibits intestinal invasion of Salmonella Typhimurium in the chicken. Bryony N. Parsons, Paul Wigley, Hannah L. Simpson, Jonathan M. Williams, Suzie Humphrey, Anne-Marie Salisbury, Alastair J. M. Watson, Stephen C. Fry, David O'Brien, Carol L Roberts, Niamh O'Kennedy, Åsa V. Keita, Johan D. Söderholm, Jonathan M. Rhodes and Barry J. Campbell.

Supporting Information File S4:

Table S4: Water soluble non-starch polysaccharide preparation derived from plantain (*Musa* AAB (Horn)), in powder format, containing in addition up to 45% plantain-derived maltodextrin carrier, and nature-equivalent colours and flavours

**Source material**	Plantain flour (Ecuador)
**Added ingredients**	Fungamyl® (Novozymes); Food grade starch-degrading enzyme (α-amylase), fully deactivated
**Nutritional values**	
Total carbohydrate	83.2 g/100g
of which sugars (maltodextrin, expressed as glucose)	40.4 g/100g
of which starch	< 0.1 g/100g
of which complex polysaccharides	40.9 g/100g
Dietary fibre (AOAC)	1.9 g/100g
Protein	<0.1 g/100g
Fat	< 0.1 g/100g
Ash	10.7 g/100g
Moisture	5.4 g/100g
Energy	1377 kJ/100g or 324 kcal/100g
Calcium	0.24 g/100g
Potassium	0.98 g/100g
Magnesium	0.11 g/100g
Sodium	3.3 g/100g
**Physical properties**	
Texture	Fine particles, flour-like consistency
Solubility	Water soluble, alcohol insoluble
Bulk density	175 g/L
Particle size	50 – 100 μm
