# Supplementary material for: Dietary Supplementation with Soluble Plantain Non-Starch Polysaccharides Inhibits Intestinal Invasion of Salmonella Typhimurium in the Chicken
Source: PLoS One. 2014 Feb 3;9(2):e87658. doi: 10.1371/journal.pone.0087658 (PMC3911995; doi:10.1371/journal.pone.0087658)

Dietary supplementation with soluble plantain non-starch polysaccharides inhibits intestinal invasion of Salmonella Typhimurium in the chicken. Bryony N. Parsons, Paul Wigley, Hannah L. Simpson, Jonathan M. Williams, Suzie Humphrey, Anne-Marie Salisbury, Alastair J. M. Watson, Stephen C. Fry, David O'Brien, Carol L Roberts, Niamh O'Kennedy, Åsa V. Keita, Johan D. Söderholm, Jonathan M. Rhodes and Barry J. Campbell.

Supporting Information File S5:

Figure S5: Desalting of Q-Sepharose anion-exchange chromatography fractions from soluble plantain NSP. Following preparative anion-exchange chromatography, sodium chloride-eluted acidic polysaccharides **(A)** and unbound neutral polysaccharide **(B)** fractions were desalted into water using multiple PD MidiTrap™ G-10 gravity columns. Carbohydrate content of eluted fractions was measured by the phenol-sulphuric acid assay (blue line). Columns were pre-calibrated with the low molecular size marker phenol red (354 Da), measured as A_{560} (red line). Arrows indicate totally included (Vt) column volume. The solid bar indicates the elution fractions collected for lyophilisation and bioassay.

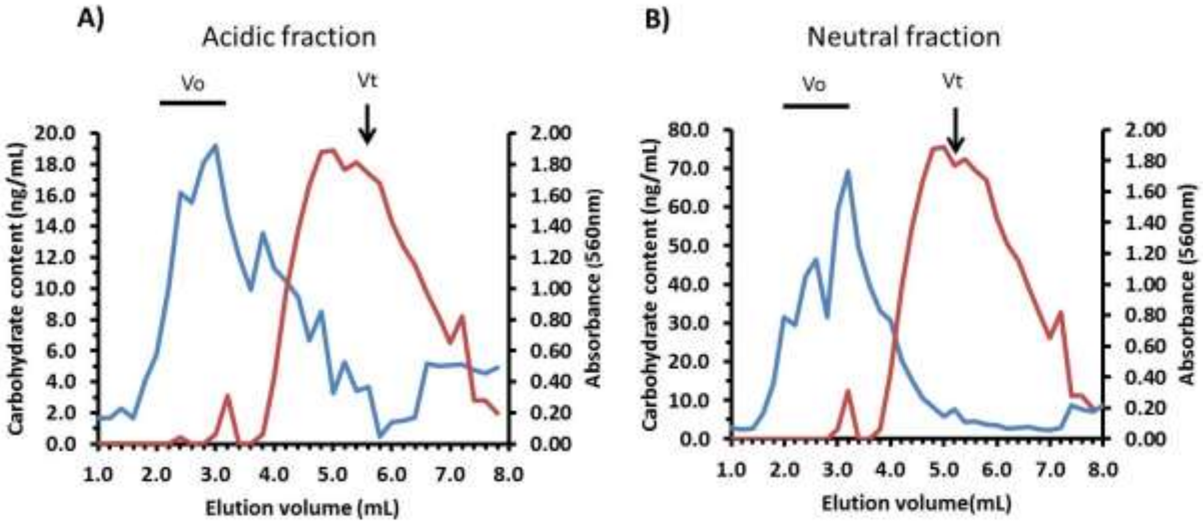

Supplement: File S5 — Contains: Figure S5: Desalting of Q-Sepharose anion-exchange chromatography fractions from soluble plantain NSP. Following preparative anion-exchange chromatography, sodium chloride-eluted acidic polysaccharides (A) and unbound neutral polysaccharide (B) fractions were desalted into water using multiple PD MidiTrap™ G-10 gravity columns. Carbohydrate content of eluted fractions was measured by the phenol-sulphuric acid assay (blue line). Columns were pre-calibrated with the low molecular size marker phenol red (354 Da), measured as A 560 (red line). Arrows indicate totally included (Vt) column volume. The solid bar indicates the elution fractions collected for lyophilisation and bioassay. (PDF) [file pone.0087658.s005.pdf]
